# Is vision-related functional burden associated with urinary incontinence?: The United States National Health and Nutrition Examination Survey 2003 to 2008

**DOI:** 10.1097/MD.0000000000042911

**Published:** 2025-06-13

**Authors:** Cheng Yan, Hong-Hai Wu, Ting-Ting Liu, Fang-Long Li, Hong-Lu Song

**Affiliations:** a Department of Clinical Pharmacy, Bethune International Peace Hospital, Shijiazhuang, Hebei, China; b Department of Urology, Bethune International Peace Hospital, Shijiazhuang, Hebei, China; c Department of Ophthalmology, Bethune International Peace Hospital, Shijiazhuang, Hebei, China.

**Keywords:** NHANES, urinary incontinence, visual burden

## Abstract

Visual impairment and urinary incontinence (UI) are 2 common diseases in the general population that can significantly impact one’s life, with multiple common risk factors. But the association between functional vision and UI is not yet clear. This study estimated the relationship between vision-related functional burden and UI in a nationally representative sample in the United States. Data from the National Health and Nutrition Examination Survey (NHANES) 2003 to 2008 were analyzed, which included 15,955 American adults aged 20 and older. Urinary incontinence and vision-related functional burden were measured separately through the NHANES questionnaire. Logistic regression was used to explore the association between vision-related functional burden and UI. A total of 9396 NHANES participants had complete data on functional vision, UI, and selected covariates. Among the participants, 11.44% (10.57, 12.37) had visual related functional burden, and the prevalence of UI was stress UI: 24.28% (23.28, 25.30), urge UI: 19.01% (17.80, 20.27), mixed urinary incontinence: 9.46% (8.86, 10.10), respectively. After controlling for age, gender, race, smoking status, drinking behavior, body mass index, hypertension, diabetes, poverty to income ratio, employment status, and education level, vision-related functional burden was still significantly associated with UI. The adjusted odds ratio (aOR) of stress UI was 1.79 (1.45–2.21, *P* < .001), the aOR of urge UI was 1.44 (1.23–1.69, *P* < .001), and the aOR of mixed urinary incontinence was 1.66 (1.32–2.10, *P* < .001). In the United States, vision-related functional burden is associated with an increased incidence of UI in adults aged 20 and above.

## 1. Introduction

More than 300 million people worldwide suffer from vision loss. Cataract, glaucoma, ametropia, macular degeneration, diabetes retinopathy, and optic neuropathy are the most common causes of vision loss.^[1–5]^ Visual function provides humans with the ability to detect their surrounding environment. In addition to increasing the risk of harmful events such as falls, visual dysfunction may also increase the incidence of orientation disorders, confusion, inability to perform daily life tasks, poor mental health, and social isolation.^[6–8]^ Individuals with low vision feel that their functional status and quality of life are significantly impaired.

Urinary incontinence (UI) refers to the involuntary leakage of urine through the urethra. According to Irwin et al’s study, approximately 13.1% of women and 5.4% of men have experienced UI.^[9]^ More importantly, a persistent UI can significantly reduce the quality of life of patients. At the same time, UI will also increase the economic burden on individuals and society. Research shows that age, body mass index (BMI), diabetes, smoking, and hypertension are all risk factors for UI.^[10–12]^ We noticed that in some research data on rest home, UI and visual impairment have a certain incidence rate,^[13,14]^ but there is no research on the association between them.

Visual function typically refers to the representation of components of the visual system. In this study, functional vision is defined as the ability to perform visual tasks in daily life in real-world scenarios based on National Health and Nutrition Examination Survey (NHANES) data. If a person expresses difficulty with any specific visual related daily life task in the NHANES questionnaire, they are considered to have functional visual impairment or visual burden.^[5,6,8]^ This study aims to use data from the NHANES from 2003 to 2008 to investigate the association between vision-related functional burden (VRFB) and UI in adults aged 20 and older in the United States (US).

## 2. Methods

### 2.1. Study population

**Figure 1.**
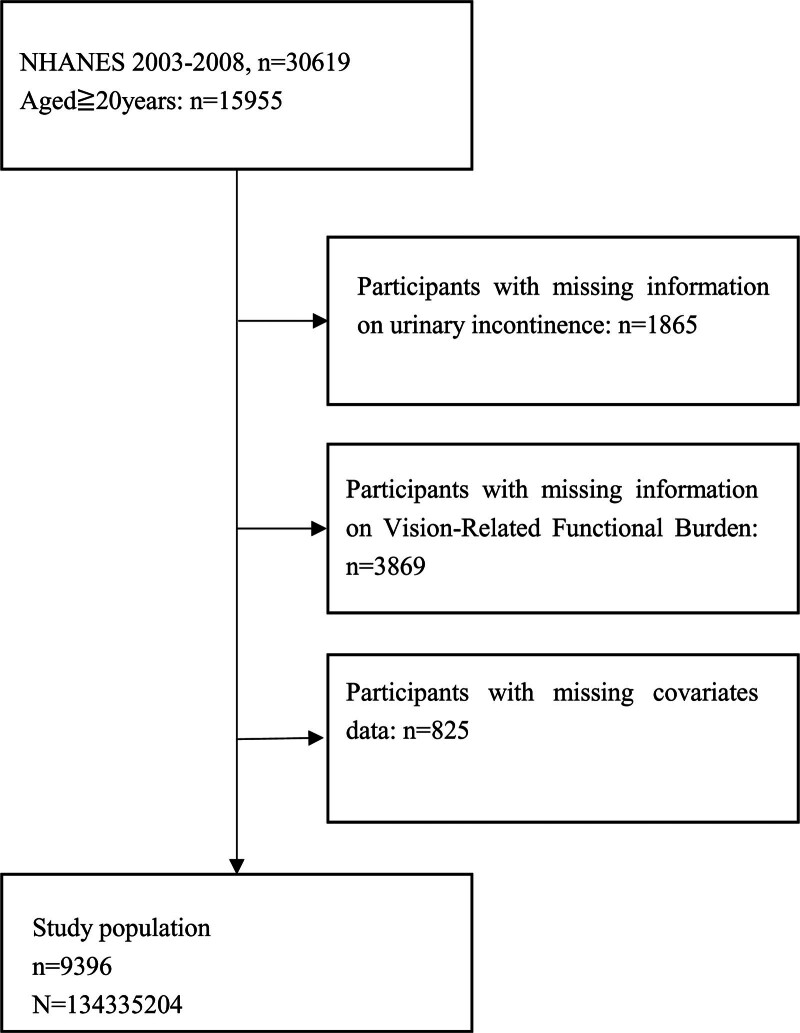
Flow chart of the screening process for the selection of eligible participants in NHANES 2003 to 2008.

NHANES is a continuous longitudinal survey dedicated to evaluating US health and nutritional status. The NHANES study was approved by the National Centre for Health Statistics Ethics Review Board in the US. Written consents were obtained from all adult participants before participation in the survey. In this study, we used public data from 3 consecutive NHANES cycles (2003–2004, 2005–2006, and 2007–2008), it is because vision-related functional burden data only exists in these 3 cycles. We chose individuals aged 20 and above as the study population because the questionnaires regarding vision-related functional burden and UI in NHANES are only targeted at adults aged 20 and above. Those under 19 years old did not fill out the relevant questionnaires. A total of 15,955 persons 20 years and older underwent a series of standardized interviews and examinations, which included demographic data, physical examination information, socioeconomic, and health-related issues. Participants who had complete data on vision-related functional burden, UI, and selected covariates were finally included (Fig. 1). Given that the NHANES adopts a stratified multistage sampling design, weighted data were required for analysis. This study sample can be extrapolated to a total of 134,335,204 individuals in the USA after applying sample weights provided by the NHANES dataset.

### 2.2. Evaluation of urinary incontinence

Trained health technicians from NHANES used established criteria to determine the presence of UI within the preceding 12 months through 2 questions. Stress urinary incontinence (SUI) was queried with, “Have you experienced urine leakage during activities such as coughing, lifting, or exercise? ” Urge urinary incontinence (UUI) was assessed with, “Have you experienced urine leakage due to an urgent sensation or pressure to urinate, but were unable to reach a restroom in time? ” Affirmative responses indicated the presence of the respective type of UI. Mixed urinary incontinence (MUI) was diagnosed when both SUI and UUI symptoms were present.^[15,16]^

### 2.3. Evaluation of functional vision

The main exposure variable for this study was whether there were any functional difficulties from the vision section (abbreviated “VIQ”), which provided personal interview data on several vision topics in the NHANES questionnaire data. Six questions were asked to evaluate the degree of visual functional difficulties, including (1) reading ordinary newsprint, (2) doing close work or chores, (3) seeing steps or curbs in dim light, (4) noticing objects to the side, (5) finding an object on a crowded shelf, and (6) driving in the daytime in a familiar place. Each question was graded on a 5-point scale: none, little, moderate, extreme, and unable to do due to eyesight. Participants were considered to have difficulty with a particular task if they reported moderate to extreme difficulty or were unable to complete the activity due to vision problems. Participants with vision-related functional burden were defined as those who reported moderate or worse in any of the 6 questions.^[5,8]^

### 2.4. Covariates

Potential confounders included demographic data: age, gender, race, education level, poverty to income ratio and employment status; health-related behaviors (smoking status, drinking behavior); BMI; systemic diseases (diabetes and hypertension). Information about age, gender, race, education level, and poverty to income ratio was derived from the NHANES demographic data module. Information about BMI was derived from the NHANES examination data module. The NHANES questionnaire data module provided information on the remaining variables. Smoking status: “Smoked at least 100 cigarettes in life?” Drinking behavior: “Had at least 12 alcohol drinks/1 year?” Hypertension: “Have you ever been told by a health professional that you had hypertension?” Diabetes: “Have you ever been told by a health professional that you have diabetes or sugar diabetes?” Employment status: “Were you working at a job or business last week?.”

### 2.5. Statistical analysis

This study performed data analysis using the Empower software (www.empowerstats.com; X&Y solutions, Inc., Boston, MA) and SPSS software, version 27.0 (SPSS, Inc., Chicago, IL). The NHANES sample weights had been applied to all estimates of the study. Continuous variables were expressed as mean ± standard deviation, and the difference test between groups was calculated by a weighted linear regression model. Meanwhile, categorical variables were expressed as percentage, and the difference test of groups was calculated by weighted chi-square test. Logistic regression models were applied to estimate the independent correlation between vision-related functional burden and UI before or after adjustment of confounders. Odds ratio (OR) and 95% confidence intervals were calculated. A two-sided *P* < .05 was considered statistically significant.

## 3. Results

### 3.1. Participants’ characteristics

A total of 9396 NHANES participants had complete data on functional vision, UI and selected covariates. Among the participants, 11.44% (10.57, 12.37) had visual related functional burden, and the prevalence of UI was SUI: 24.28% (23.28, 25.30), UUI: 19.01% (17.80, 20.27), MUI: 9.46% (8.86, 10.10), respectively. Table 1 showed the characteristics of all UI patients and non-UI patients. Compared to individuals without UI, participants with UI were older and had a higher BMI (*P* < .05). Compared to men, women had a higher proportion of UI(*P* < .05). There were significant racial/ethnic differences between participants with and without SUI and UUI (*P* < .05). There was also a statistically significant difference in drinking habits, education level, poverty to income ratio, and employment status between adults with UI and those without UI (*P* < .05), however, no statistical difference in education level and poverty to income ratio was observed between SUI and non-SUI adults (*P* = .657/0.310). The statistically significant difference in smoking habits was only observed between UUI and non-UUI adults (*P* < .05). The prevalence of hypertension and diabetes in adults with UI was higher than that in adults without UI (*P* < .05). The prevalence of VRFB in participants with UI was higher than that in non-UI participants (*P* < .05).

**Table 1:** Baseline characteristics of UI group versus the non-UI group.

Characteristics	Non-SUI	SUI	*P*-value	Non-UUI	UUI	*P*-value	Non-MUI	MUI	*P*-value
n*	7180	2216		7356	2040		8455	941	
Age†	46.73 (45.89, 47.58)	52.68 (51.61, 53.75)	**<.001**	46.18 (45.36, 47.01)	56.67 (55.50, 57.84)	**<.001**	47.34 (46.50, 48.17)	56.21 (54.99, 57.44)	**<.001**
BMI	28.23 (27.95, 28.51)	29.71 (29.26, 30.15)	**<.001**	28.30 (27.98, 28.62)	29.81 (29.50, 30.12)	**<.001**	28.42 (28.12, 28.71)	30.24 (29.70, 30.78)	**<.001**
Gender			**<.001**			**<.001**			**<.001**
Female	37.34 (35.93, 38.76)	90.93 (89.42, 92.24)		46.08 (44.96, 47.20)	68.54 (66.11, 70.88)		46.43 (45.37, 47.49)	87.86 (84.93, 90.28)	
Male	62.66 (61.24, 64.07)	9.07 (7.76, 10.58)		53.92 (52.80, 55.04)	31.46 (29.12, 33.89)		53.57 (52.51, 54.63)	12.14 (9.72, 15.07)	
Race			**<.001**			**<.001**			.082
Other race	5.06 (4.24, 6.02)	4.64 (3.46, 6.21)		5.31 (4.42, 6.37)	3.47 (2.51, 4.76)		5.11 (4.32, 6.04)	3.50 (2.01, 6.03)	
Mexican American	6.96 (5.56, 8.68)	5.87 (4.69, 7.31)		7.01 (5.64, 8.69)	5.34 (4.02, 7.05)		6.86 (5.53, 8.49)	5.06 (3.61, 7.05)	
Other Hispanic	3.55 (2.47, 5.06)	3.29 (2.38, 4.55)		3.54 (2.52, 4.95)	3.23 (2.14, 4.85)		3.47 (2.46, 4.88)	3.62 (2.33, 5.58)	
Non-Hispanic White	73.84 (69.97, 77.37)	78.69 (74.82, 82.11)		74.87 (71.15, 78.26)	75.63 (71.19, 79.58)		74.63 (70.90, 78.04)	78.67 (73.68, 82.94)	
Non-Hispanic Black	10.60 (8.43, 13.24)	7.51 (5.80, 9.66)		9.26 (7.32, 11.66)	12.33 (9.78, 15.44)		9.92 (7.86, 12.46)	9.15 (6.99, 11.90)	
Education level			.657			**<.001**			**<.001**
Above high school	58.86 (55.87, 61.77)	58.82 (54.90, 62.63)		60.01 (56.99, 62.97)	53.87 (50.09, 57.60)		59.40 (56.47, 62.27)	53.53 (48.96, 58.03)	
High school or GED	24.82 (23.20, 26.52)	25.66 (23.26, 28.23)		24.76 (23.05, 26.54)	26.19 (24.21, 28.27)		24.93 (23.35, 26.58)	25.97 (22.91, 29.29)	
Less than high school	16.32 (14.54, 18.27)	15.52 (13.05, 18.37)		15.23 (13.51, 17.12)	19.94 (16.81, 23.50)		15.67 (13.95, 17.55)	20.50 (16.80, 24.77)	
Poverty to income ratio			.310			**<.001**			**<.001**
≧339%	50.01 (47.19, 52.82)	48.09 (44.07, 52.14)		50.86 (47.84, 53.86)	43.94 (39.94, 48.03)		50.41 (47.51, 53.31)	41.20 (36.12, 46.47)	
131%–338%	34.23 (32.16, 36.37)	35.71 (32.90, 38.63)		34.04 (31.81, 36.34)	36.94 (34.57, 39.37)		34.27 (32.19, 36.41)	37.65 (33.82, 41.64)	
≦130%	15.76 (14.34, 17.30)	16.20 (13.78, 18.94)		15.10 (13.57, 16.78)	19.12 (16.65, 21.87)		15.32 (13.87, 16.89)	21.15 (17.71, 25.04)	
Drinking behavior			**<.001**			**<.001**			**<.001**
No	22.29 (20.18, 24.54)	32.40 (29.23, 35.75)		23.68 (21.42, 26.09)	29.28 (26.28, 32.46)		23.67 (21.48, 26.01)	35.03 (30.84, 39.47)	
Yes	77.71 (75.46, 79.82)	67.60 (64.25, 70.77)		76.32 (73.91, 78.58)	70.72 (67.54, 73.72)		76.33 (73.99, 78.52)	64.97 (60.53, 69.16)	
Hypertension			**<.001**			**<.001**			**<.001**
No	70.40 (68.53, 72.20)	59.66 (56.72, 62.54)		70.96 (69.08, 72.77)	54.31 (51.59, 57.01)		69.38 (67.59, 71.12)	52.58 (48.94, 56.19)	
Yes	29.60 (27.80, 31.47)	40.34 (37.46, 43.28)		29.04 (27.23, 30.92)	45.69 (42.99, 48.41)		30.62 (28.88, 32.41)	47.42 (43.81, 51.06)	
Diabetes or pre-diabetes			**<.001**			**<.001**			**<.001**
No	91.35 (90.40, 92.22)	87.52 (85.07, 89.63)		92.06 (90.87, 93.11)	83.45 (80.89, 85.73)		91.18 (90.16, 92.10)	83.19 (79.33, 86.45)	
Yes	8.65 (7.78, 9.60)	12.48 (10.37, 14.93)		7.94 (6.89, 9.13)	16.55 (14.27, 19.11)		8.82 (7.90, 9.84)	16.81 (13.55, 20.67)	
Employment status			**<.001**			**<.001**			**<.001**
Unemployed	29.84 (27.67, 32.10)	42.81 (39.69, 45.98)		29.43 (27.55, 31.39)	48.14 (44.71, 51.59)		31.21 (29.19, 33.31)	49.99 (45.60, 54.38)	
Employed	70.16 (67.90, 72.33)	57.19 (54.02, 60.31)		70.57 (68.61, 72.45)	51.86 (48.41, 55.29)		68.79 (66.69, 70.81)	50.01 (45.62, 54.40)	
Smoking behavior			.217			**.015**			.434
No	49.62 (47.25, 51.99)	51.50 (48.70, 54.28)		50.82 (48.38, 53.26)	46.90 (44.47, 49.35)		50.19 (47.99, 52.38)	48.98 (45.99, 51.99)	
Yes	50.38 (48.01, 52.75)	48.50 (45.72, 51.30)		49.18 (46.74, 51.62)	53.10 (50.65, 55.53)		49.81 (47.62, 52.01)	51.02 (48.01, 54.01)	
Difficulty reading ordinary newsprint			**<.001**			**<.001**			**<.001**
No	95.30 (94.65, 95.88)	91.65 (90.01, 93.05)		95.07 (94.27, 95.76)	91.64 (90.07, 92.98)		94.83 (94.07, 95.49)	90.50 (87.97, 92.54)	
Yes	4.70 (4.12, 5.35)	8.35 (6.95, 9.99)		4.93 (4.24, 5.73)	8.36 (7.02, 9.93)		5.17 (4.51, 5.93)	9.50 (7.46, 12.03)	
Difficulty doing close work or chores			**<.001**			**<.001**			**<.001**
No	95.76 (95.17, 96.28)	91.73 (89.98, 93.20)		95.32 (94.69, 95.89)	92.47 (91.34, 93.46)		95.20 (94.64, 95.72)	90.74 (88.21, 92.77)	
Yes	4.24 (3.72, 4.83)	8.27 (6.80, 10.02)		4.68 (4.11, 5.31)	7.53 (6.54, 8.66)		4.80 (4.28, 5.36)	9.26 (7.23, 11.79)	
Difficulty seeing steps or curbs in dim light			**<.001**			**<.001**			**<.001**
No	96.49 (95.78, 97.08)	91.02 (89.38, 92.42)		96.24 (95.59, 96.79)	90.57 (88.85, 92.04)		95.97 (95.33, 96.53)	87.36 (84.44, 89.80)	
Yes	3.51 (2.92, 4.22)	8.98 (7.58, 10.62)		3.76 (3.21, 4.41)	9.43 (7.96, 11.15)		4.03 (3.47, 4.67)	12.64 (10.20, 15.56)	
Difficulty noticing objects to the side			**<.001**			**<.001**			**<.001**
No	98.05 (97.72, 98.33)	95.86 (94.73, 96.76)		98.05 (97.70, 98.34)	95.28 (94.08, 96.24)		97.90 (97.57, 98.19)	93.87 (91.91, 95.38)	
Yes	1.95 (1.67, 2.28)	4.14 (3.24, 5.27)		1.95 (1.66, 2.30)	4.72 (3.76, 5.92)		2.10 (1.81, 2.43)	6.13 (4.62, 8.09)	
Difficulty finding an object on a crowded shelf			**<.001**			**<.001**			**<.001**
No	97.97 (97.64, 98.25)	95.59 (94.25, 96.63)		97.81 (97.41, 98.15)	95.62 (94.44, 96.56)		97.68 (97.25, 98.04)	94.68 (92.73, 96.13)	
Yes	2.03 (1.75, 2.36)	4.41 (3.37, 5.75)		2.19 (1.85, 2.59)	4.38 (3.44, 5.56)		2.32 (1.96, 2.75)	5.32 (3.87, 7.27)	
Difficulty driving in the daytime in a familiar place			**<.001**			**.001**			**<.001**
No	99.09 (98.86, 99.27)	97.97 (97.13, 98.56)		99.02 (98.70, 99.26)	97.95 (97.02, 98.60)		98.98 (98.68, 99.21)	97.26 (95.75, 98.25)	
Yes	0.91 (0.73, 1.14)	2.03 (1.44, 2.87)		0.98 (0.74, 1.30)	2.05 (1.40, 2.98)		1.02 (0.79, 1.32)	2.74 (1.75, 4.25)	
Vision-related functional burden			**<.001**			**<.001**			**<.001**
No	90.52 (89.65, 91.32)	82.45 (79.98, 84.67)		90.14 (89.22, 90.99)	81.83 (79.70, 83.79)		89.60 (88.70, 90.43)	78.64 (75.09, 81.81)	
Yes	9.48 (8.68, 10.35)	17.55 (15.33, 20.02)		9.86 (9.01, 10.78)	18.17 (16.21, 20.30)		10.40 (9.57, 11.30)	21.36 (18.19, 24.91)	

*P* < .05 is marked in bold.

BMI = body mass index, MUI = mixed urinary incontinence, SUI = stress urinary incontinence, UI = urinary incontinence, UUI = urge urinary incontinence.

* n represents unweighted number, and the remaining values are weighted values using NHANES MEC examination weight.

† Figures are expressed as mean ± standard error (for mean age, BMI), other figures are expressed as percent (95% confidence intervals).

The characteristics of all 9396 self-reported participants with and without VRFB were shown in Table 2. Compared with individuals without VRFB, adults with VRFB were older(age 53.93 [52.77, 55.09] vs 47.73 [46.54, 48.33], *P* < .05) and had higher BMI (29.24 [28.79, 29.69] vs 28.50 [28.22, 28.79], *P* < .05). Compared to men, women had a higher percentage of VRFB (*P* < .05). There was no statistically significant difference in race/ethnicity between participants with and without VRFB (*P* = .9985). The prevalence of hypertension and diabetes was higher in adults with VRFB (*P* < .05). There was a statistically significant difference in smoking status, education level, poverty to income ratio, and employment status between adults with VRFB and those without VRFB (*P* < .05), however, no statistical difference in drinking behavior was observed between VRFB and non-VRFB adults (*P* = .400). Compared to participants without VRFB, those with VRFB had a higher incidence of any type of UI (*P* < .05).

**Table 2:** Baseline characteristics of vision-related functional burden group versus the non-vision-related functional burden group.

Characteristics	Non-vision-related functional burden	Vision-related functional burden	*P*-value
n*	8150	1246	
Age†	47.43 (46.54, 48.33)	53.93 (52.77, 55.09)	**<.001**
BMI	28.50 (28.22, 28.79)	29.24 (28.79, 29.69)	**.003**
Gender			**<.001**
Female	49.66 (48.69, 50.64)	55.63 (52.47, 58.74)	
Male	50.34 (49.36, 51.31)	44.37 (41.26, 47.53)	
Race			.999
Other race	4.98 (4.14, 5.98)	4.80 (3.33, 6.87)	
Mexican American	6.69 (5.41, 8.25)	6.70 (4.83, 9.23)	
Other Hispanic	3.50 (2.50, 4.86)	3.39 (2.09, 5.45)	
Non-Hispanic White	75.01 (71.11, 78.54)	75.08 (70.79, 78.94)	
Non-Hispanic Black	9.83 (7.81, 12.30)	10.02 (7.71, 12.93)	
Education level			**<.001**
Above high school	60.06 (57.16, 62.89)	49.43 (44.42, 54.45)	
High school or GED	24.72 (23.28, 26.22)	27.37 (24.00, 31.03)	
Less than high school	15.21 (13.43, 17.19)	23.19 (20.38, 26.26)	
Poverty to income ratio			**<.001**
≧339%	51.24 (48.30, 54.17)	36.42 (32.19, 40.87)	
131%–338%	34.20 (32.12, 36.34)	37.61 (33.54, 41.86)	
≦130%	14.56 (13.20, 16.04)	25.97 (21.94, 30.46)	
Drinking behavior			.400
No	24.60 (22.35, 27.01)	25.81 (22.77, 29.10)	
Yes	75.40 (72.99, 77.65)	74.19 (70.90, 77.23)	
Hypertension			**<.001**
No	69.39 (67.57, 71.15)	55.44 (51.51, 59.30)	
Yes	30.61 (28.85, 32.43)	44.56 (40.70, 48.49)	
Diabetes or pre-diabetes			**<.001**
No	91.46 (90.48, 92.34)	82.44 (78.81, 85.56)	
Yes	8.54 (7.66, 9.52)	17.56 (14.44, 21.19)	
Employment status			**<.001**
Unemployed	30.92 (28.97, 32.93)	49.01 (45.66, 52.37)	
Employed	69.08 (67.07, 71.03)	50.99 (47.63, 54.34)	
Smoking behavior			**<.001**
No	51.65 (49.53, 53.77)	37.87 (33.89, 42.02)	
Yes	48.35 (46.23, 50.47)	62.13 (57.98, 66.11)	
SUI			**<.001**
No	77.40 (76.15, 78.60)	62.76 (59.39, 66.00)	
Yes	22.60 (21.40, 23.85)	37.24 (34.00, 40.61)	
UUI			**<.001**
No	82.44 (81.12, 83.68)	69.82 (66.93, 72.56)	
Yes	17.56 (16.32, 18.88)	30.18 (27.44, 33.07)	
MUI			**<.001**
No	91.60 (90.88, 92.26)	82.34 (79.79, 84.63)	
Yes	8.40 (7.74, 9.12)	17.66 (15.37, 20.21)	

*P* < .05 is marked in bold.

BMI = body mass index, MUI = mixed urinary incontinence, SUI = stress urinary incontinence, UI = urinary incontinence, UUI = urge urinary incontinence.

* n represents unweighted number, and the remaining values are weighted values using NHANES MEC examination weight.

† Figures are expressed as mean ± standard error (for mean age, BMI), other figures are expressed as percent (95% confidence intervals).

### 3.2. Association between VRFB and UI

Table 3 showed the adjusted odds ratio (aOR) and 95% confidence interval of UI determined by VRFB. In model 1, according to the multivariate logistic regression analysis after adjusting for age, gender, and race, the aOR of VRFB in patients with UI was significantly higher than that in patients without UI. In model 2, after further adjustment of smoking status, drinking behavior and BMI, the aOR of SUI was 1.80 (1.46–2.22, *P* < .05), the aOR of UUI was 1.55 (1.33–1.81, *P* < .05), and the aOR of MUI was 1.82 (1.47–2.26, *P* < .05). In model 3, after adjustment of age, gender, race, smoking status, drinking behavior, BMI, hypertension, diabetes, education level, poverty to income ratio, and employment status, the aOR of SUI was 1.79 (1.45–2.21, *P* < .05), the aOR of UUI was 1.44 (1.23–1.69, *P* < .05), and the aOR of MUI was 1.66 (1.32–2.10, *P* < .05). In general, VRFB was significantly associated with any type of UI.

**Table 3:** Multivariate logistic regression model of the relationship between vision-related functional burden and urinary incontinence.

	SUI												UUI												MUI											
	aOR	95% CI		*P*	aOR	95% CI		*P*	aOR	95% CI		*P*	aOR	95% CI		*P*	aOR	95% CI		*P*	aOR	95% CI		*P*	aOR	95% CI		*P*	aOR	95% CI		*P*	aOR	95% CI		*P*
Status	Model 1				Model 2				Model 3				Model 1				Model 2				Model 3				MODEL 1				Model 2				Model 3			
Impaired functional vision																																				
No/a little difficulty	1 [Reference]				1 [Reference]				1 [Reference]				1 [Reference]				1 [Reference]				1 [Reference]				1 [Reference]				1 [Reference]				1 [Reference]			
Difficulty reading ordinary newsprint	1.85	1.47	2.33	<.001	1.72	1.34	2.21	<.001	1.71	1.32	2.22	**<.001**	1.40	1.10	1.78	.010	1.31	1.02	1.70	.045	1.18	0.90	1.55	.234	1.64	1.20	2.23	.003	1.52	1.09	2.12	.018	1.33	0.93	1.91	.131
Difficulty with up-close work or chores	2.00	1.49	2.70	<.001	1.95	1.44	2.64	<.001	1.94	1.44	2.63	**<.001**	1.32	1.09	1.60	.008	1.28	1.05	1.56	.020	1.19	0.96	1.46	.118	1.66	1.22	2.28	.003	1.60	1.17	2.20	.006	1.45	1.04	2.04	**.038**
Difficulty seeing steps or curbs in dim light	1.94	1.47	2.57	<.001	1.80	1.36	2.39	<.001	1.78	1.34	2.36	**<.001**	1.84	1.44	2.35	<.001	1.73	1.35	2.22	<.001	1.57	1.23	2.02	**.001**	2.33	1.75	3.11	<.001	2.16	1.61	2.90	<.001	1.93	1.43	2.60	**<.001**
Difficulty noticing objects to side	1.98	1.38	2.82	.001	1.93	1.33	2.80	.001	1.90	1.30	2.76	**.002**	1.82	1.30	2.54	.001	1.79	1.28	2.50	.002	1.57	1.13	2.17	**.012**	2.41	1.64	3.54	<.001	2.34	1.58	3.45	<.001	1.99	1.33	2.98	**.002**
Difficulty finding object on crowded shelf	2.49	1.80	3.43	<.001	2.43	1.74	3.38	<.001	2.40	1.73	3.33	**<.001**	1.60	1.17	2.18	.005	1.57	1.16	2.13	.007	1.39	1.02	1.91	**.048**	2.02	1.27	3.22	.005	1.97	1.25	3.12	.006	1.69	1.05	2.71	**.038**
Difficulty driving in the daytime in familiar place	1.48	0.84	2.60	.180	1.53	0.87	2.68	.147	1.48	0.85	2.57	.173	1.04	0.60	1.79	.895	1.07	0.62	1.83	.820	0.90	0.53	1.54	.700	1.48	0.80	2.73	.221	1.49	0.82	2.73	.202	1.23	0.66	2.29	.512
Vision-related functional burden	1.91	1.55	2.35	<.001	1.80	1.46	2.22	<.001	1.79	1.45	2.21	**<.001**	1.62	1.40	1.89	<.001	1.55	1.33	1.81	<.001	1.44	1.23	1.69	**<.001**	1.94	1.58	2.38	<.001	1.82	1.47	2.26	<.001	1.66	1.32	2.10	**<.001**

Model 1: adjusted for age, gender, and race.

Model 2: Adjusted for age, gender, race, smoking behavior, drinking behavior, BMI.

Model 3: Adjusted for age, gender, race, smoking behavior, drinking behavior, BMI, hypertension, diabetes, poverty to income ratio, employment status and education level.

*P* < .05 is marked in bold in Model 3.

aOR = adjusted odds ratio, BMI = body mass index, MUI = mixed urinary incontinence, SUI = stress urinary incontinence, UI = urinary incontinence, UUI = urge urinary incontinence.

The research results of associations between VRFB and UI stratified by gender/age/race were shown in Table 4. The associations between VRFB and UI in females were all significant(*P* < .05). In the study population aged 60+, VRFB was also significantly associated with any type of UI (*P* < .05). Research on racial stratification showed that there was a significant association between VRFB and any type of UI among non-Hispanic White and non-Hispanic Black (*P* < .05).

**Table 4:** Subgroup analysis of the association of UI subtype (SUI, UUI, and MUI) and vision-related functional burden.

	SUI				UUI				MUI			
	OR	95% CI		*P*-value	OR	95% CI		*P*-value	OR	95% CI		*P*-value
Gender												
Male	1.46	0.90	2.37	.124	1.71	1.35	2.17	**<.001**	1.88	1.19	2.98	**.012**
Female	1.87	1.48	2.37	**<.001**	1.31	1.05	1.64	**.025**	1.59	1.26	2.01	**<.001**
Age												
20–39	2.31	1.37	3.90	**.004**	1.47	0.84	2.56	.196	2.48	1.22	5.03	**.015**
40–59	1.71	1.23	2.37	**.003**	1.34	1.04	1.71	**.028**	1.47	1.00	2.16	.052
60+	1.46	1.13	1.89	**.005**	1.51	1.12	2.06	**.012**	1.59	1.11	2.28	**.016**
Race												
Mexican American	1.65	1.09	2.49	**.026**	1.26	0.76	2.09	.385	1.59	0.91	2.79	.119
Other Hispanic	1.43	0.56	3.64	.420	0.77	0.36	1.67	.523	0.95	0.27	3.28	.924
Non-Hispanic White	1.76	1.38	2.23	**<.001**	1.35	1.11	1.64	**.005**	1.58	1.22	2.05	**.002**
Non-Hispanic Black	1.70	1.27	2.28	**.001**	2.10	1.56	2.82	**<.001**	2.15	1.45	3.20	**.001**
Other race	3.67	1.31	10.31	**.022**	2.65	0.72	9.80	.159	2.69	0.65	11.07	.185

All data were adjusted for gender (except gender-specific estimates), age (except age-specific estimates), race(except race-specific estimates), smoking behavior, drinking behavior, BMI, hypertension, diabetes, poverty to income ratio, employment status and education level.

*P* < .05 is marked in bold.

BMI = body mass index, MUI = mixed urinary incontinence, SUI = stress urinary incontinence, UI = urinary incontinence, UUI = urge urinary incontinence.

## 4. Discussion

This cross-sectional study examined the association between VRFB and UI in adults aged 20 and older in the United States. The results indicated a significant association between VRFB and UI using 6 years nationally representative data from NHANES. Furthermore, after adjusting for gender, race, age, smoking status, drinking behavior, BMI, hypertension, diabetes, education level, poverty to income ratio, and employment status, this association was still significant. Due to the large sample size and reasonable quality control, our analysis should be reliable.

By comparing the data in Table 1 and Table 2, we found that VFRB and UI population have a higher age and BMI, a higher proportion of women and unemployed personnel, and a higher prevalence of diabetes and hypertension than healthy people. These findings are similar to some previous research results.^[5,15,17,18]^In Table 3, we evaluated the association between VRFB and UI. Self-reported functional difficulties secondary to vision problems were assessed over multiple categories, including (1) reading ordinary newsprint, (2) doing close work or chores, (3) seeing steps or curbs in dim light, (4) noticing objects to the side, (5) finding an object on a crowded shelf, and (6) driving in the daytime in a familiar place. From our results, it could be seen that 5 out of 6 visual impairments were significantly associated with SUI, 3 with UUI, and 4 with MUI. Difficulty seeing steps or curbs in dim light, difficulty noticing objects to side, and difficulty finding object on crowded shelf was associated with any types of UI (*P* < .05). This suggests that we should pay more attention to the visual functional burden of the 3 forms mentioned above.

To our knowledge, this is the first study to explore the association between VRFB and UI. UI can seriously affect the quality of life of patients through SUI, UUI, or both. SUI is the involuntary loss of urine with increased intra-abdominal pressure or physical exertion (such as coughing, jumping, and physical exercise), while UUI is caused by stimulation or loss of neural control over bladder contraction.^[15,16]^ Although the causal relationship between VRFB and UI is not yet clear, there are several possible reasons. We speculate that the mechanisms linking VRFB to UI may be related to the following factors: First, patients with visual impairments may feel troubled by their inability to see their surroundings clearly, and their movements may become slower. This could cause anxiety after feeling the urge to urinate, and they may also be unable to quickly locate the restroom, resulting in urinary leakage; second, individuals with impaired or lost vision often cannot independently engage in necessary basic activities and daily living activities. The long-term persistence of this pattern may be related to changes in mood, manifested as inferiority and weakness, as well as unwillingness to engage in various activities. Furthermore, reduced activity may be associated with obesity and metabolic syndrome. Although it has not yet been fully clarified, the effect of metabolic syndrome on the bladder may be achieved by acting on the metabolically active urothelium, and the metabolically active urothelium may be affected by the direct inflammatory effect of autonomic nervous supply, atherosclerosis induced ischemia, or a combination of these 2 mechanisms,^[17–19]^ which may be related to the occurrence of UI.

Women and the elderly are susceptible populations to UI,^[11,16,19]^ our research had also observed this. VRFB was significantly associated with any type of UI in women, individuals aged 60 and above, non-Hispanic White and non-Hispanic Black populations, indicating that the association between VRFB and UI should be taken seriously in the above-mentioned groups, necessary early education and intervention should be carried out. Addressing health issues in high-risk populations is challenging, often due to limited opportunities for health education and screening. Public health guidance is crucial in addressing these challenges. Healthcare professionals should actively participate in the health management of high-risk populations to reduce the risk of complications and improve patients’ quality of life.

This study has some limitations that should be acknowledged. First, NHANES in the United States is a cross-sectional survey. It does not provide longitudinal follow-up data. Due to its retrospective nature, further research is needed to demonstrate the causal relationship between VRFB and UI. Second, certain unknown or unmeasured variables, which could potentially confound the relationships studied, were not part of the dataset. Therefore, it is necessary to collect more comprehensive data in future research. Third, Urinary incontinence, metabolic syndrome, and vision-related functional burden were identified by individual’s responses to questionnaires, inaccurate reporting or recall bias may confound the study results.

## 5. Conclusions

In summary, this study showed that the vision-related functional burden of adults aged 20 and above in the United States was associated with an increased incidence of UI. Although the causal relationship was not yet clear, the possibility of UI among patients with vision-related functional burden should also be considered. Clinical doctors should screen for visual impairments and intervene early in UI patients.

## Author contributions

**Conceptualization:** Hong-Hai Wu.

**Data curation:** Cheng Yan.

**Formal analysis:** Ting-Ting Liu.

**Investigation:** Cheng Yan.

**Methodology:** Ting-Ting Liu.

**Resources:** Cheng Yan, Hong-Lu Song.

**Software:** Fang-Long Li.

**Supervision:** Fang-Long Li.

**Validation:** Cheng Yan, Fang-Long Li.

**Visualization:** Cheng Yan, Hong-Lu Song.

**Writing – original draft:** Cheng Yan.

**Writing – review & editing:** Cheng Yan, Hong-Lu Song.
